# The Effectiveness of Assistive Technologies for Older Adults and the Influence of Frailty: Systematic Literature Review of Randomized Controlled Trials

**DOI:** 10.2196/31916

**Published:** 2022-04-04

**Authors:** Marina Liselotte Fotteler, Viktoria Mühlbauer, Simone Brefka, Sarah Mayer, Brigitte Kohn, Felix Holl, Walter Swoboda, Petra Gaugisch, Beate Risch, Michael Denkinger, Dhayana Dallmeier

**Affiliations:** 1 DigiHealth Institute Neu-Ulm University of Applied Sciences Neu-Ulm Germany; 2 Research Unit on Ageing Agaplesion Bethesda Clinic Ulm Ulm Germany; 3 Institute for Geriatric Research Ulm University Ulm Germany; 4 Geriatric Center Ulm/Alb-Donau Ulm Germany; 5 Institute for Medical Information Processing, Biometry, and Epidemiology Ludwig Maximilian University of Munich Munich Germany; 6 Institute for Global Health Sciences University of California San Francisco San Francisco, CA United States; 7 Fraunhofer-Institute for Industrial Engineering Stuttgart Germany; 8 Department of Epidemiology Boston University School of Public Health Boston, MA United States

**Keywords:** assistive technology, older adults, systematic review, frailty

## Abstract

**Background:**

The use of assistive technologies (ATs) to support older people has been fueled by the demographic change and technological progress in many countries. These devices are designed to assist seniors, enable independent living at home or in residential facilities, and improve quality of life by addressing age-related difficulties.

**Objective:**

We aimed to evaluate the effectiveness of ATs on relevant outcomes with a focus on frail older adults.

**Methods:**

A systematic literature review of randomized controlled trials evaluating ATs was performed according to the PRISMA (Preferred Reporting Items for Systematic Reviews and Meta-Analyses) guidelines. The Ovid Medline, PsycINFO, SocIndex, CINAHL (Cumulative Index to Nursing and Allied Health Literature), CENTRAL (Cochrane Central Register of Controlled Trials), and IEEEXplore databases were searched from January 1, 2009, to March 15, 2019. ATs were included when aiming to support the domains autonomy, communication, or safety of older people with a mean age ≥65 years. Trials performed within a laboratory setting were excluded. Studies were retrospectively categorized according to the physical frailty status of participants.

**Results:**

A total of 19 trials with a high level of heterogeneity were included in the analysis. Six device categories were identified: mobility, personal disease management, medication, mental support, hearing, and vision. Eight trials showed significant effectiveness in all or some of the primary outcome measures. Personal disease management devices seem to be the most effective, with four out of five studies showing significant improvement of disease-related outcomes. Frailty could only be assessed for seven trials. Studies including participants with significant or severe impairment showed no effectiveness.

**Conclusions:**

Different ATs show some promising results in well-functioning but not in frail older adults, suggesting that the evaluated ATs might not (yet) be suitable for this subgroup. The uncertainty of the effectiveness of ATs and the lack of high-quality research for many promising supportive devices were confirmed in this systematic review. Large studies, also including frail older adults, and clear standards are needed in the future to guide professionals, older users, and their relatives.

**Trial Registration:**

PROSPERO CRD42019130249; https://www.crd.york.ac.uk/prospero/display_record.php?RecordID=130249

## Introduction

Advancements in medicine and public health have led to a rise in life expectancy and are among the main reasons for the changing demographic structure in many countries. In the European Union, the share of people aged 65 years and over is projected to rise by almost 31 million (or 7%) until 2040, while the overall population is estimated to decrease by approximately 1 million [1]. The growing number of older citizens, often with multiple morbidities, leads to an increased demand for health care services and professionals [2]. Coupled with rising costs for diagnosis and treatment, politicians and stakeholders anticipate difficulties in providing adequate care in the near future. One essential approach is to empower older adults to manage their own health and remain independent as long and extensive as possible [3].

The use of assistive technologies (ATs) in older persons’ care has been fueled by these developments, helping to maintain seniors’ autonomy, safety, or communication at home or in residential facilities [4-8]. Thus, ATs may not only increase older adults’ quality of life (QoL) but also contribute to a relief of health care systems and, in particular, formal and informal caregivers [2]. In recent years, a variety of devices addressing problems associated with, for example, dementia [9-11], hypertension [12,13], Parkinson disease [14,15], and loneliness [16] have entered the market. In the literature, the term AT is used to include, among others, telemedical applications [17,18], robotics [4,19], virtual reality [20,21], and sensors [22], but can also cover more conventional technologies such as hearing or vision aids [23,24]. The lack of a uniform definition and the resulting heterogeneity preclude harmonized recommendations, guidance, and structured research [4,25-27]. Despite a large amount of existing literature on the use of ATs for older people, the effectiveness of these devices remains unclear [3,18,28-30]. Users, as well as their formal and informal caregivers, are often overwhelmed by the different options, and up-to-date guidance from insurance companies or other institutions is lacking [31-33].

Previous research has shown that, so far, AT is not likely to replace personal care but rather to supplement it [34]. Ideally, older adults should be able to use ATs with no or little help or supervision to avoid adding workload to the caregiver [35]. In particular, frail older adults with increased dependency could benefit from AT. However, this population often expresses a mixed attitude toward ATs and needs special support when using these devices [36]. The process of becoming a regular user of AT as an older adult is complex [3,36-38]. Usability, the ease of integration into daily life, access and affordability, and individual aspirations and characteristics are some factors influencing the use of AT among older adults [29,38,39].

In this study, we systematically reviewed randomized controlled trials (RCTs) to provide a synthesis of high-quality evidence on the effectiveness of ATs for nonfrail and frail older adults. In this context frailty is defined as “a state of increased vulnerability to poor resolution of homeostasis following a stress, which increases the risk of adverse outcomes including falls, delirium and disability” [40]. It has been previously suggested that frailty also firmly relates to functional status [41]. We defined the effectiveness of ATs as the capability to positively impact issues related to autonomy, communication, and/or safety. These three areas of impact were chosen by an expert committee within the project Future City Ulm 2030, which aims to design a holistic and sustainable urban environment with the inclusion of digital solutions such as ATs. RCTs are widely considered to be the gold standard for effectiveness research, providing the highest level of evidence for causality [42]. The analysis in this review was based on this concept. Three research questions were defined:

RQ1: What are the primary measures used to evaluate ATs?

RQ2: What types of ATs have effectively influenced autonomy, communication, and/or safety in adults aged 65 years and older?

RQ3: What influence does frailty have on the effectiveness of an AT?

## Methods

### Design

A systematic literature review was performed using the guidelines from the PRISMA (Preferred Reporting Items for Systematic Reviews and Meta-Analyses) statement [43] (see the PRISMA Checklist in Multimedia Appendix 1). The analysis was based on a protocol published in the PROSPERO register under registration number CRD42019130249.

### Search Strategy

The following databases were searched: Ovid Medline, PsycINFO, SocIndex, CINAHL (Cumulative Index to Nursing and Allied Health Literature), CENTRAL (Cochrane Central Register of Controlled Trials), and IEEEXplore. The search string was composed of three parts, focusing on age, methodology, and technology, respectively, combined by the operator AND. The three parts were (1) a previously published search filter for geriatric medicine [44], adapted slightly for the purpose of this study; (2) a sensitivity- and precision-maximizing version of the Cochrane RCT filter in the Ovid format [45]; and (3) a string for technology developed with experts and terms used for AT identified through other related systematic reviews [28,37,46]. The complete search string in the Ovid syntax is provided in Multimedia Appendix 2; the string was adapted to fit the requirements of other databases. Searches were performed on March 15, 2019, and all records were imported to the web-based software Covidence for screening. Reference lists of the selected studies and other systematic reviews on the topic were screened for additional records.

### Inclusion and Exclusion Criteria

Eligible for inclusion were peer-reviewed studies published in English or German between January 1, 2009, and March 15, 2019, reflecting the momentum that research on the effectiveness of AT has gained in the last decade. The date restriction was the only filter used in the database search. We included technologies that can assist with issues regarding autonomy, communication, or safety. Other inclusion criteria were (1) a study population with a mean age of 65 years or higher; (2) the study design being an RCT, including a control group with no intervention, an alternative intervention, or a placebo device; (3) the home of the senior, a residential facility, a nursing home, or similar as the study setting; and (4) any sort of technical, socioeconomic, ethical, or medical outcome measuring the impact of the technology on stakeholders (eg, patients, relatives, nurses, physicians).

Exclusion criteria were (1) studies performed in a laboratory setting; (2) studies analyzing robotics, virtual reality, telemedicine, or lifestyle interventions or technologies for rehabilitative or therapeutic purposes; (3) technologies demanding regular involvement of formal or informal caregivers; and (4) applications that have to be used in periodic training units. These exclusion criteria were selected to focus the analysis on technologies that are affordable and usable for the target population in their daily life without external support from relatives, caregivers, or medical staff.

### Data Extraction and Analysis

Two authors (MLF and VM) independently screened all records and the studies selected for full-text analysis. Discrepancies were discussed and a third person was consulted, if necessary, until consensus was reached. Data extraction was carried out independently by both authors. The effectiveness of devices was recorded by extracting outcome data and statistical significance for primary outcome measures (*P*<.05). RCTs with a crossover design were individually analyzed for potential carryover effects by three authors (MLF, VM, and MD) (see Multimedia Appendix 3). If a serious impact was expected, only the first part of the study until the crossover was considered to ensure comparability with noncrossover trials. In semicrossover or delayed-start trials, where the control group switches to the intervention after a predefined period, only the first study phase was analyzed, making such studies identical to RCTs with a parallel-group design.

In cases of missing data, authors were contacted via email up to twice. The study population’s frailty status was categorized retrospectively according to their functional level into one of the four following categories: not impaired/independent (nonfrail), slightly impaired (prefrail), significantly impaired, and severely impaired/disabled (frail) [41]. A risk of bias (RoB) analysis was performed according to the Cochrane RoB tool to judge the quality of the selected studies [47]. Funding and the recruitment process were also assessed. Due to the heterogeneity of interventions and outcomes, it was not possible to perform a meta-analysis. A qualitative synthesis and a narrative review were performed to interpret study results and draw conclusions. To identify additional insights, subgroups according to frailty status and device category were considered. Figures were created using Microsoft PowerPoint and Excel for Mac Version 16.35.

## Results

### Included Studies

**Figure 1:**
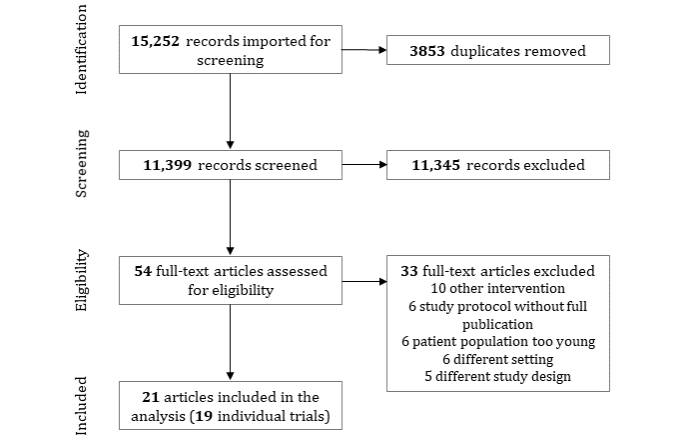
PRISMA (Preferred Reporting Items for Systematic Reviews and Meta-Analyses) diagram for the study selection process.

After removal of duplicates, the search yielded 11,399 records. No articles were identified through other sources as described above. A total of 54 full texts were assessed for eligibility, 21 of which were included in the review (Figure 1). Reasons for exclusion of full texts were (1) the kind of intervention (such as the evaluation of training sessions or the use of therapeutic devices; n=10), (2) a study protocol without full publication (n=6), (3) the patient population being too young (n=6), (4) a different setting (mostly laboratory, n=6), and (5) a different study design (n=6) (also see Multimedia Appendix 4). The 21 records covered 19 individual trials with a total study population of 1768 participants.

### Description of Studies

Table 1 summarizes data on the design and participants of the 19 studies included in the analysis. The articles were published between 2010 and 2018. The trend shows an increase in research output across 2017 and 2018, the years with the highest number of publications respectively (n=4). Overall, most studies were conducted in Europe (n=10), followed by five studies from the United States. Among the 19 studies, 10 were confirmatory RCTs and the rest were pilot or feasibility RCTs. Most studies employed a regular parallel-group design. Two studies were conducted using a delayed-start/semicrossover approach [23,48] and five studies employed a crossover design [8,11,14,24,27]. Of those five, two trials were judged to have a low risk of carryover, one studying an electronic vision enhancement system [24] and the other evaluating the benefit of video calls versus regular phone calls for patients with dementia [8].

Having a mean age ≥65 years as an inclusion criterion for our search, there were still large differences in the inclusion criteria at the study level: ≥18 years in three studies [13,14,24], 45-90 years in one study [17], 55-79 years in one study [49], ≥60 years in one study [11], and ≥65 years in six studies [7,23,50-53]. The other seven trials did not have age as an inclusion criterion but targeted conditions present specifically in older adults, such as cardiovascular conditions, dementia, or being a senior housing resident [6,8,12,22,27,48,54]. Table 1 provides the mean (SD) age for each study stratified by intervention and control group.

Most studies had participants’ homes as their study site (n=14). The investigation period varied from 1 month [8,11,14,17] to 12 months [7,22,23]. The largest trial included 203 study participants [52]. The mean ages of study populations ranged from 68.9 years to 87.8 years. Only one study assessed frailty at baseline based on the Fried Frailty Score [53]. Frailty could be estimated retrospectively for six other studies [7,14,22,23,50,54]. On average, the frailty levels were found to be slightly impaired/prefrail (n=4), significantly impaired/frail (n=1), and severely impaired/frail (n=1) (Table 2).

**Table 1:** Overview of included studies, describing the study design and participants.

Study	Year	Country	Study design				Study participants		
			Study type	Group design	Setting	Participants randomized, n		Age (years), mean (SD)	
								IG^a^	CG^b^
Scheffer et al [52]	2012	Netherlands	Full	Parallel group	Home	203		80.8 (9.0)	81.2 (9.3)
Mira et al [50]	2014	Spain	Full	Parallel group	Home	102		70.9 (8.0)	72.9 (6.0)
Hägglund et al [54]	2015	Sweden	Full	Parallel group	Home	82		75.0 (8.0)	76.0 (7.0)
Humes et al [49]	2017	United States	Full	Parallel group	Home	163		68.9 (5.9)	69.5 (6.7)
Rantz et al [22]	2017	United States	Full	Parallel group	Nursing home	171		83.6 (9.4)	86 (8.0)
Ong et al [51]	2018	Singapore	Full	Parallel group	Home	197		77.0	77.0
Levine et al [48]	2016	United States	Full	Delayed-start	Home	54		71.5 (12.2)	70.5 (10.5)
Adrait et al [23]; Nguyen et al [55]	2017	France	Full	Delayed-start	Home	51		83.0 (6.2)	82.3 (7.2)
Elston et al [14]	2010	United Kingdom	Full	Crossover^c^	Home	42		71.5 (11.3)	70.4 (8.7)
Bray et al [24]; Taylor et al [56]	2017	United Kingdom	Full	Crossover^d^	Home	100		69.79 (19.97)	72.94 (16.63)
Tchalla et al [7]	2013	France	Pilot	Parallel group	Home	96		87.8 (6.5)	85.3 (6.3)
Goldstein et al [17]	2014	United States	Pilot	Parallel group	Home	60		69.0 (10.6)	69.6 (11.3)
Lam et al [12]	2016	United States	Pilot	Parallel group	Home	134		68.9 (13.2)	71.1 (13.0)
Or and Tao [13]	2016	Hong Kong	Pilot	Parallel group	Home	63		69.3 (9.7)	69.7 (10.2)
Lauriks et al [6]	2018	Netherlands	Pilot	Parallel group	Nursing home	54		84.3 (5.6)	83.1 (7.1)
Schoon et al [53]	2018	Netherlands	Pilot	Parallel group	Home and nursing home	86		79.9 (5.5)	80.9 (7.0)
Brath et al [27]	2013	Austria	Pilot	Crossover^c^	Home	77		69.4 (4.8)	69.4 (4.8)
Davison et al [11]	2015	Australia	Pilot	Crossover^c^	Nursing home	16		86.0 (5.2)	86.0 (5.2)
Van der Ploeg et al [8]	2016	Australia	Pilot	Crossover^d^	Nursing home	17		86.7 (range 83.0-93.0)	86.7 (range 83.0- 93.0)

^a^IG: intervention group.

^b^CG: control group.

^c^Crossover study with expected carryover effect.

^d^Crossover study without expected carryover effect.

**Table 2:** Frailty assessment.

Study	Frailty	
	Scale	Frailty status^a^
Mira et al [50]	Barthel ADL^b^	Slightly impaired (prefrail)
Hägglund et al [54]	Short-Form 36 Physical	Slightly impaired (prefrail)
Rantz et al [22]	Gait speed^c^	Severely impaired (frail)
Adrait et al [23]; Nguyen et al [55]	Lawton-IADL^d^	Significantly impaired (frail)
Elston et al [14]	Gait speed	Slightly impaired (prefrail)
Tchalla et al [7]	Lawton-IADL^e^	Slightly impaired (prefrail)
Schoon et al [53]	Fried Frailty Score	18.6% of participants frail at baseline

^a^Categorized according to a method proposed by Brefka et al [41], except for Schoon et al [53].

^b^ADL: activities of daily living.

^c^Collection of ADL and IADL also mentioned with no data reported but provided by the authors upon request.

^d^IADL: instrumental activities of daily living.

^e^Timed-Up-and-Go test also performed with inconclusive results.

### Types of ATs and Effectiveness

#### Overview

The 19 selected trials evaluated devices representing the following six domains: (1) mobility (n=5 [6,7,14,52,53]), (2) personal disease management (n=5 [13,22,48,51,54]), (3) medication (n=4 [12,17,27,50]), (4) mental support (n=2 [8,11]), (5) hearing (n=2 [23,49]), and (6) vision (n=1 [24]). All devices addressed at least one of the areas of autonomy, safety, or communication. An overlap was noticeable in the categories mobility and medication with devices targeting both autonomy and safety issues (Table 2). Interventions, controls, and primary outcomes studied in the included trials are presented in Table 2.

#### Mobility

Significant effectiveness was only reported in a pilot study for a nightlight path, which reduced falls among older people classified as slightly impaired/prefrail who had mild and moderate Alzheimer disease (odds ratio 0.73, 95% CI 0.15-0.88) [7]. Home automatization for people with dementia living in group homes [6], a mobile safety alarm with a drop sensor for community-dwelling older persons [52], and a gait-speed monitoring and feedback device for older people at risk for falling [53] were not effective. In a crossover study on the use of a metronome to improve QoL in individuals classified as slightly impaired/prefrail who were suffering from Parkinson disease, no evidence of effectiveness could be shown. The authors reported the possible impact of a carryover effect, which we agree with. Unfortunately, separate data were not reported for the first part of the study and could not be obtained from the authors [14].

#### Personal Disease Management

A system consisting of a tablet computer connected to a patient scale was effective for participants classified as slightly impaired/prefrail who had heart failure. Both primary endpoints, the effect on self-care behavior and health-related QoL, improved in the intervention group after a 90-day trial. System adherence was high with a median of 88% (IQR 78%-96%) [54]. In a semicrossover trial of a device reminding participants suffering from type 2 diabetes to perform self-monitoring of blood glucose, between-group comparison did not show improved levels of glycated hemoglobin. However, participants in the intervention group experienced a statistically significant decrease. Furthermore, the intervention group missed 6% of their measures and the control group missed 22% of measures, representing a statistically significant difference [48]. In another study, a medical alert protection system for older persons living alone was found to be effective in reducing the length of stay for hospital admissions. However, the number of emergency department visits and hospitalizations could not be significantly reduced [51]. In a pilot RCT evaluating the effect of a tablet computer–based self-monitoring system for older people suffering from type 2 diabetes and/or hypertension, systolic blood pressure was significantly more reduced in the intervention group compared with the control group. No significant differences were observed for diastolic blood pressure, blood glucose, glycated hemoglobin, chronic disease knowledge, and monitoring frequency. The within-group comparison showed a significant improvement of diastolic blood pressure in the intervention group (∆=–5.7, 95% CI –9.3 to –2.2). Approximately 30% (9/33) of participants in the intervention group reported technical problems [13]. An environmentally embedded sensor system for early illness alerts was not effective for a severely impaired/frail population [22].

#### Medication

A study of a tablet-based app for medication self-management reported a significant improvement in adherence as well as the number of missed doses (27.3% reduction in the intervention group) in a slightly impaired/prefrail population. A reduction of medication errors was only found for patients with a higher error rate prior to the study. Although the mean satisfaction score with the AT in the intervention group was high (8.5 out of 10), 59% (30/51) of intervention group participants required assistance using the AT and almost 12% (6/51) stated that the device did not help at all [50]. In a trial evaluating a “talking pill bottle,” informing hypertensive adults with low health literacy about the correct administration and dosage of their medication, no between-group effect but a significant reduction in blood pressure within the intervention group was reported. Additionally, a vast majority of participants found the device easy to use (63/68, 93%) and many agreed that it helped them to understand (77%) and correctly take their medication (74%) [12]. Two telemedical medication reminders (smartphone and pillbox) did not improve medication adherence [17]. A crossover trial of electronic blisters with an expected carryover did not report significant results [27].

#### Mental Support

A multimedia device with personalized music, videos, messages, and pictures installed by family members was tested in a pilot sample of 11 nursing home residents. Almost half of the participants needed assistance operating the device due to limited sensory or cognitive abilities. Nevertheless, staff and family members agreed they would recommend the AT for residents with dementia. During the 2-month crossover period, depression and anxiety were significantly reduced in the intervention group. However, a carryover effect seems likely, and no data are available for the precrossover phase of the study [11]. In the second study, video calls with family members were not effective in reducing agitation in nursing home residents with dementia [8]. A retrospective analysis of frailty was not possible for the studies evaluating ATs for mental support.

#### Hearing

Humes et al [49] compared the best-practice service for hearing aids to an over-the-counter and a placebo device, and found that both the best-practice and the over-the-counter device did effectively benefit participants. Only participants testing the best-practice device showed greater satisfaction than the placebo group. No differences in usage (hours/day) were detected among the groups [49]. In another RCT, hearing aids did not significantly improve dementia-related symptoms or QoL in older adults classified as significantly impaired/frail or benefit their caregivers [23,55].

#### Vision

A portable electronic vision enhancement system was compared to conventional optical magnifiers in a crossover trial that was published in two articles [24,56]. The authors did not report separate data for the first study phase before the crossover. However, a carryover effect was not expected in this study. Near-vision visual function was significantly improved (∆=0.57, 95% CI 0.33-0.81) [24]. Although reading speed did not significantly increase when using the portable device, the researchers significantly associated the accessibility of smaller print sizes and the ease of carrying out other tasks with the portable device. When considering frequency of use, the study participants seemed to prefer optical low-vision aids to the electronic system (unstandardized effect size estimate –0.93, 95% CI –1.29 to –0.57) [56]. An economic evaluation was also performed, and the authors concluded that the AT was a cost-effective way to improve near-vision visual function with an incremental cost-effectiveness ratio of US $997.12 (95% CI US $651.89-2066.92) per unit. Improvements in QoL did not prove cost-effective [24]. A retrospective analysis of frailty was not possible for these studies evaluating ATs supporting vision.

### Evaluation of Risk of Bias of the Included Studies

**Figure 2:**
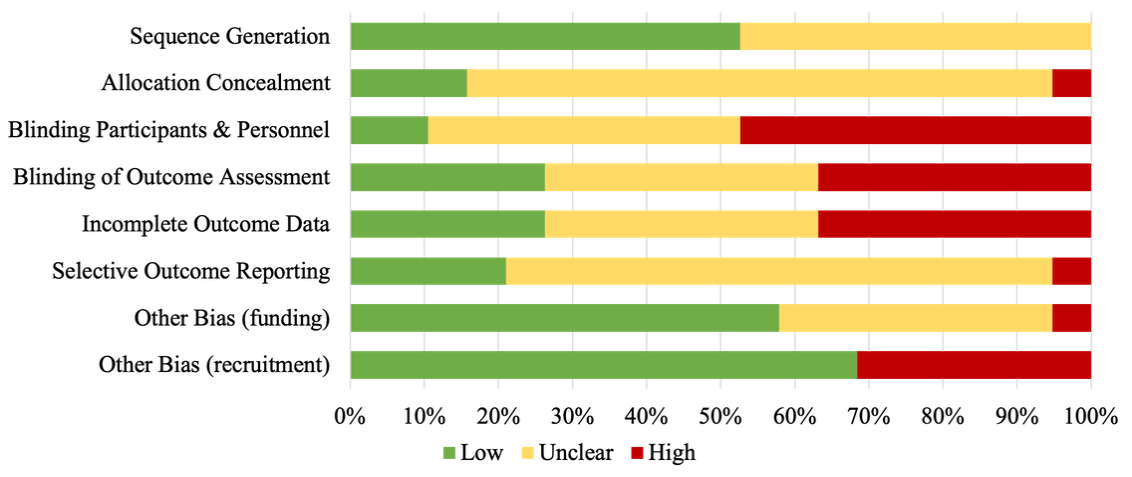
Judgment of risk of bias categories for each included study presented as percentages across all included studies.

**Figure 3:**
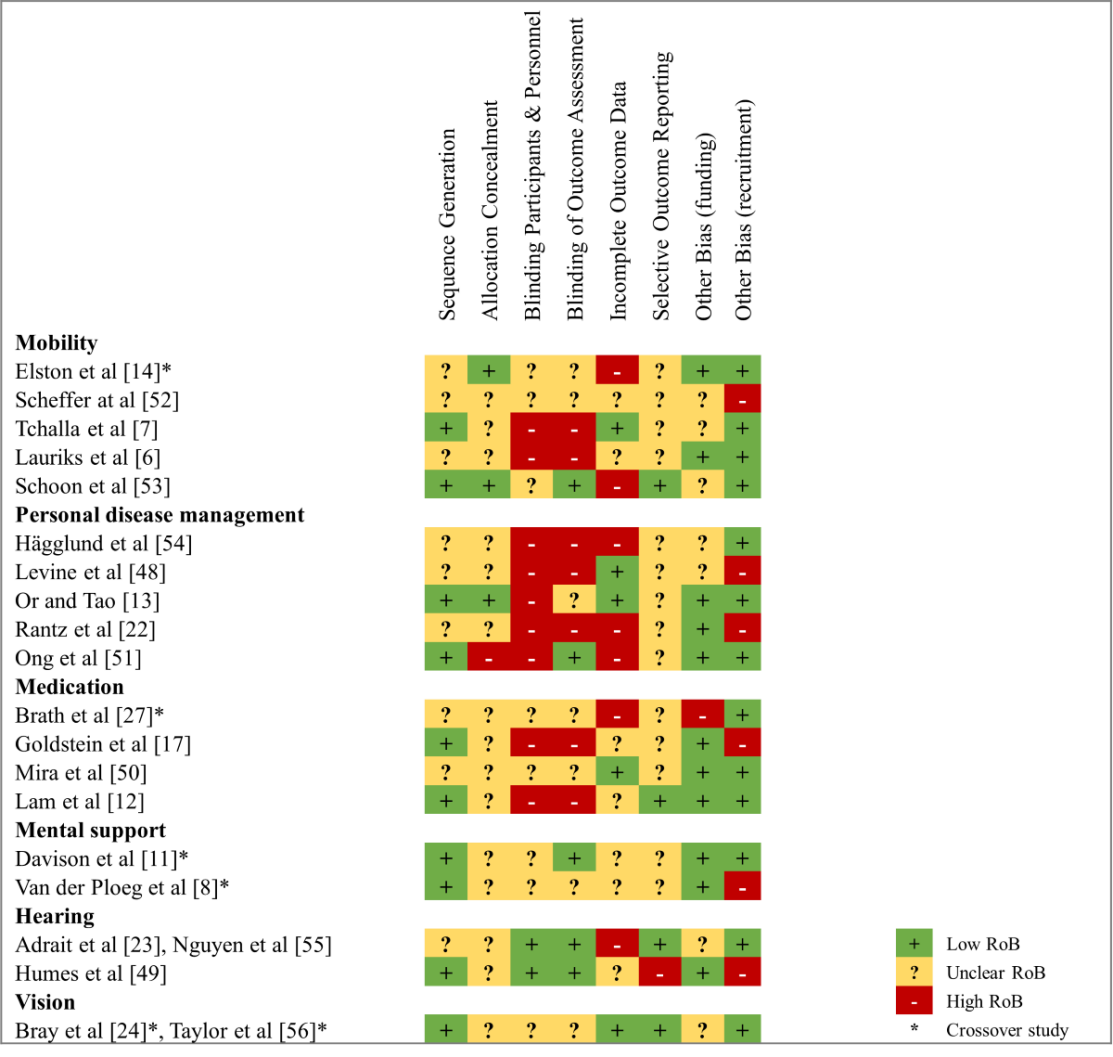
Judgment of risk of bias categories for each included study, ordered by assistive technology category and publication year.

Figure 2 and Figure 3 show the results of the RoB analysis. In four categories, we considered more than 30% of the studies to have a high RoB due to issues in blinding of participants and personnel, blinding of outcome assessment, incomplete outcome data, and recruitment bias. The crossover studies had a lower RoB. In three studies, analyzing a medication self-management app [50], electronic vision enhancement system [24,56], and multimedia device for people with dementia [11], no category was judged to have a high RoB (Figure 3). All studies had incomplete reporting for at least one category. A full RoB assessment was therefore not possible.

From the available information, it appears that testing an AT often purports difficulties with blinding participants and personnel. Nevertheless, unblinded studies are considered to have a higher RoB. In six studies, outcome assessors were not blinded, although it would have been possible [6,7,12,17,22,48,54]. These studies were thus judged to have a high RoB. Several studies had missing data or skewed dropout rates, and were thus considered to be at high RoB for incomplete outcome reporting [14,22,23,27,51,53,54]. The recruitment process was deemed to be sufficient in most studies. For six studies, it was judged that there was a high risk for the study population not being representative of the target population [8,17,22,48,49,52]. Only one study was judged to have a high RoB due to funding. The rationale for this decision was that the manufacturing company of the devices tested was the study sponsor and had a major influence on the study design [27].

### Outcome Assessment

A total of 70 primary outcome measures were extracted from the 19 trials (Table 3 and Multimedia Appendix 5). ATs were evaluated using measures focusing on efficacy (n=30), functionality (n=8), mental status (n=8), QoL (n=7), health-related impact (eg, knowledge, behavior; n=5), usability (n=5), effect on caregivers (n=5), and economic aspects (n=2). The two trials with two publications each reported the largest diversity of measures with five (QoL, functionality, mental status, health impact, caregivers) [23,55] and four (QoL, efficacy, usability, economic) [24,56] outcome categories covered, respectively. The highest overall number of primary outcomes was collected by two studies with eight measures, respectively [6,22].

**Table 3:** Overview of interventions, domain(s) of interest, and outcomes studied in the included trials.

Study		Intervention	Control	Domain(s) of interest				(Primary) outcome(s)^a^
				A^b^	S^c^	C^d^		
**Mobility**								
	Elston et al [14]	Metronome for the improvement of QoL^e^ in people with Parkinson disease	Usual medication	✓	✓		Parkinson disease mobility, QoL	
	Scheffer et al [52]	Mobile safety alarm with a drop sensor for community-dwelling older persons	No mobile safety alarm	✓	✓		Frequency of going outside	
	Tchalla et al [7]	Nightlight path for patients with Alzheimer disease	No nightlight path		✓		Fall incidence	
	Lauriks et al [6]	Assistive home technology for people with dementia living in group homes	No assistive home technology		✓		QoL (self-rated, observed by caregiver); assessment of need for older persons; number and location of fall incidents; use of restraints; caregiver job satisfaction, workload, and general health	
	Schoon et al [53]	Gait speed monitoring and feedback device for older people at risk for falling	No gait speed monitoring	✓	✓		Subjective general health and mental well-being; number of weekly measurements (compliance); fall incidence; incidence of injurious falls; fear of falling	
**Personal disease management**								
	Hägglund et al [54]	Home intervention system for patients with heart failure	Standard heart failure information		✓		Heart failure self-care behavior; health-related QoL	
	Levine et al [48]	Automated self-management monitor for blood glucose for low-income seniors	No automated self-management monitor for blood glucose	✓	✓		Glycated hemoglobin level; frequency of self-monitoring of blood glucose	
	Or and Tao [13]	Tablet computer–based self-monitoring system for type 2 diabetes mellitus and/or hypertension	Conventional self-monitoring method	✓	✓		Glycated hemoglobin level; fasting blood glucose level; blood pressure; diabetes/ hypertension knowledge; self-monitoring frequency	
	Rantz et al [22]	Nonwearable sensor system to monitor the status of older persons	Usual care		✓		Walking speed; GAITRite^f^; QoL; depression; mental state; ADL^g^ and IADL^h^; hand grip	
	Ong et al [51]	Medical alert protection system for older people living at home alone	Telephone follow-up		✓		Emergency department visits; number of hospitalizations; total length of stay for admitted patients	
**Medication**								
	Brath et al [27]	Mobile health–based electronic medication blisters for patients with diabetes	Standard medication blisters, routine care, handwritten medication intake diaries		✓		Medication adherence	
	Goldstein et al [17]	Telemedicine medication reminder systems: electronic pillbox, smartphone app for older adults with heart failure	Silent pillbox or silent smartphone	✓	✓		Medication adherence	
	Mira et al [50]	Medication self-management app for older adults taking multiple medications	Oral and written information on safe medication use	✓	✓		Self-perceived health status; medication adherence; medication errors; missed doses	
	Lam et al [12]	Talking pill bottle for patients with hypertension	Usual care	✓	✓		Self-efficacy for appropriate medication use; medication adherence; refill adherence; medication knowledge; blood pressure	
**Mental support**								
	Davison et al [11]	Personalized multimedia device for people with dementia	Social control: weekly 30-min visits from researchers (reading, discussing things)			✓	Agitation; depression in dementia; anxiety in dementia	
	Van der Ploeg et al [8]	Internet video chat (Skype) for nursing home residents with dementia	Landline telephone			✓	Agitation; call duration	
**Hearing**								
	Adrait et al [23]; Nguyen et al [55]	Active hearing aid for patients with Alzheimer disease	Inactive hearing aid			✓	Neuropsychiatric symptoms; IADL Alzheimer disease– related QoL; caregiver QoL; patient and caregiver health profile; Alzheimer disease cognition	
	Humes et al [49]	Best-practice hearing aid and over-the-counter models	Placebo device			✓	Hearing aid performance and benefit	
**Vision**								
	Bray et al [24]; Taylor et al [56]	Portable electronic vision enhancement system for people with visual impairments	Optical magnifiers	✓			Near-vision visual function; vision-related QoL; cost-effectiveness and cost-utility; maximum reading speed; frequency of use	

^a^If no distinction between primary and secondary outcomes was made, all outcomes are listed.

^b^A: autonomy.

^c^S: safety.

^d^C: communication.

^e^QoL: quality of life.

^f^Automatic measurement of certain variables (eg, velocity, step length) while participants walk across the GAITRite Mat.

^g^ADL: activities of daily living.

^h^IADL: instrumental activities of daily living.

Unfortunately, six outcome measures from crossover studies with expected carryover could not be analyzed due to a lack of data for the first phase of the study. Of the remaining 64 outcomes, 13 (20%) showed a significantly positive effect of the AT in the categories efficacy, usability, and QoL. However, considering the RoB, seven of those outcomes, covering all three categories, might be impacted [7,13,48,49,51,54] (Table 4). More detailed data on individual quantitative outcomes (test statistics, effect sizes, significance levels) can be found in Multimedia Appendix 5.

**Table 4:** Statistically significant outcome measures including a judgment of high risk of bias (RoB).

Outcome measure		Outcome category	Reason for high RoB	
**Bray et al [24]; Taylor et al [56]**				Not applicable (no high RoB)
	Near-vision visual function	Efficacy		
	Vision-related QoL^a^	QoL		
	Frequency of use	Usability		
	Cost-effectiveness (near-vision visual function vs carer and intervention costs)	Economic		
**Hägglund et al [54]**				No blinding; all dropouts in IG^b^
	Heart failure self-care behavior	Efficacy		
	Health-related QoL	QoL^b^		
Hearing aid performance/benefit (Humes et al [49])		Efficacy	Per-protocol analysis; recruitment through newspaper ads	
Usage frequency (Levine et al [48])		Usability	No blinding; risk of recruitment bias (people who refused to participate were older, had lower glycated hemoglobin levels, and were less likely to be African American)	
**Mira et al [50]**				Not applicable (no high RoB)
	Medication adherence	Efficacy		
	Medication errors	Efficacy		
Total length of stay for admitted patients (Ong et al [51])		Efficacy	No blinding (allocation was discussed with the participants); very high dropout rates in IG (32% vs 1% in the CG^c^), resulting in a change in the IG:CG ratio from 1:1 to 1:3	
Decrease of diastolic blood pressure (Or and Tao [13])		Efficacy	No blinding	
Fall incidence (Tchalla et al [7])		Efficacy	No blinding	

^a^QoL: quality of life.

^b^IG: intervention group.

^c^CG: Control group.

## Discussion

### Principal Findings

To our knowledge, this systematic review is the first to collect and synthesize evidence exclusively from RCTs evaluating the effectiveness of ATs for older adults in a realistic living environment (ie, no laboratory setting), taking into account participants’ frailty status. More than 11,000 records were identified from a broad range of databases with different focuses. Only 19 RCTs fulfilled the inclusion criteria. The selected trials were very heterogeneous with respect to the ATs applied as well as the outcomes, which made it difficult to summarize the evidence [57]. Our analysis did not provide strong confirmation for the overall effectiveness of AT in older adults. Only personal disease management apps seem to be promising for this population.

Many older citizens wish to remain independent and continue living at home for as long as possible [58]. The hope is that AT can support this goal, positively impacting QoL, reducing health care utilization, and relieving caregivers [2]. The results of this review suggest some effectiveness of personal disease management apps. Four of the five personal disease management trials showed a significant improvement in self-care and monitoring of health- or disease-related indicators [13,48,51,54], effectively influencing safety and, in some cases, autonomy (related to RQ2). A recent review investigated the effectiveness of mobile health apps for blood pressure management in populations with digital barriers, among other older adults. The authors confirmed the promise of ATs for chronic disease management but also emphasized the need for more studies including vulnerable populations [57]. The willingness for and success of AT-supported self-management can also be dependent on the disease [59]. This could not be confirmed, as our analysis did not provide any additional insights for the effectiveness of personal disease management when stratifying by disease.

Considering other existing research, hearing aids seem to be an effective way to improve the domain of communication in adults aged 65 years and older [49,60,61]. With respect to other devices, the study evaluating a portable vision enhancement system reported an effective improvement due to the AT, but the authors stated that no other comparable evidence supported these results [24]. Further research is needed in all categories for a more reliable assessment.

Regarding frailty of older adults (RQ3), only one study included this population characterization in their evaluation of a gait speed feedback device [53]. Although no significant effectiveness could be shown, similar compliance and success rates for frail participants were found, suggesting that this mobility-supporting device can also be appropriate for this subgroup. We were able to retrospectively estimate the frailty status for a total of 6 out of the 19 studies (Table 2). However, some instruments used might not be ideal for the estimation of frailty, as they are influenced by the underlying disease of the study population [14,23]. In studies where, on average, participants were categorized to have significant or severe impairment (frailty), the AT did not show any effectiveness. As an example, out of the five personal disease management trials, only the one including participants categorized as severely impaired/frail did not show significant results in terms of improvement [22]. Additionally, ATs were also not effective in the four studies that were conducted in nursing homes. Overall, nursing home residents are known to be more dependent, with a high prevalence of frailty [40,53].

Altogether, our results indicate that ATs might not yet suitably address the needs of frail older adults. A possible explanation is the fact that ATs are not usually developed with the specific needs of this population in mind. A recent systematic review on the use of communication technologies to improve social well-being in older adults found that more off-the-shelf products exist than devices designed specifically for older adults [62]. A qualitative study on the use of AT by frail older people showed specific needs of this subgroup when becoming users of AT, such as prescription support, training, and follow-ups [36]. This highlights that frail older adults might face specific challenges when using AT that could affect the performance of such technologies. Further research should focus more on this vulnerable group, including measures of frailty for the study populations.

We also showed that the evaluation of an AT is usually unidimensional (RQ1). Many factors, especially social, economic, or ethical aspects, are hardly investigated [29]. For example, only two studies analyzed in this review evaluated the impact of the AT for formal caregivers, showing no improvement for their working conditions or health [6,23]. Two trials considered economic aspects of ATs [22,24]. Ethical challenges have not been taken into account at all, despite their importance for data management issues and in the setting of smart housing technology [29,63].

The unclear findings on the effectiveness of ATs for older adults align with those of other systematic literature reviews on the topic [18,28-30,62]. Our strict inclusion and exclusion criteria, especially the requirements for the type of technology, mean age, and setting, resulted in the inclusion of 19 RCTs in the final analysis. Almost half of the studies included were pilot or feasibility trials. This shows that there is still a lack of research addressing the use of ATs for older adults at home or in similar settings [57,62,64]. A crossover design, where the control group changes to the intervention after a predefined period, was found to be commonly used when evaluating ATs. Possible reasons for this could be the easier recruitment as every participant can test the device, which might also lead to a reduction of dropout numbers due to an increased motivation to remain in the study. However, the average dropout rates were similar among the two RCT types (parallel design: 13.8%; crossover design: 12.6%). In this context, three studies with a regular parallel-group design reported noticeably higher dropout rates in the intervention group and were judged to have a high RoB for incomplete outcome data [51,53,54]. The retraction of consent and complexity of ATs were mentioned as possible reasons for this. Several records were excluded because they evaluated ATs in a laboratory setting. To gain insightful and reliable evidence on the actual effectiveness of AT, it is necessary to evaluate the devices being used by older persons within a realistic setting [57]. The challenges that arise in terms of ethical, economic, and logistic issues when performing studies with older adults in their own homes are part of the reason for the current lack of research [35,65].

### Limitations

There is a lack of a uniform definition concerning ATs for older people. This makes searching for and selecting suitable studies difficult, and increases the risk of missing relevant research. The search string resulted in almost 11,400 records. Only 19 were selected for the review, indicating an insufficient precision caused on the one hand by the lack of standardized terminology and on the other hand by the vast amount of existing literature evaluating AT in clinical settings rather than in the home environment. Additionally, the technologies considered in this analysis are heterogenous, thus limiting the possibilities for analysis, in particular the performance of a GRADE (Grading of Recommendations, Assessment, Development and Evaluations) assessment to rate the certainty of evidence as suggested by the Cochrane Collaboration. The number of trials per device type is not sufficient to form a definite conclusion of the effectiveness of AT. When the analysis for this review was performed, the new RoB 2 tool from the Cochrane Collaboration [66] was still undergoing pilot testing, and therefore we used the original RoB tool, first published in 2008 [47], for our analysis. Although the mean age of participants across all trials was 76.3 years, six identified trials included participants below the age of 65 years. Unfortunately, the authors did not present a stratified analysis by age, thus limiting the generalizability of the results to the older population.

### Conclusion

Researchers, politicians, and health care professionals across the globe have high hopes for AT to support older adults. Many devices are freely available on the market and are often used even though the effectiveness is not supported by current research, as shown in this review. The number of available RCTs evaluating ATs in older populations is limited and many only include a small number of study participants. Further studies with larger, well-characterized samples of older adults are necessary to allow for further stratification (eg, for frailty). Additionally, it is important to expand the focus and include economic, social, ethical, and technological aspects besides the medical outcomes. Formal and informal caregivers may, in some cases, benefit from AT even more than the older adults themselves and should therefore be included in future studies. The new Medical Devices Regulation of the European Union includes stricter controls and requires an evaluation of all medical devices before certification. In this context, our review intends to add value by identifying the current gaps in the literature, emphasizing the importance of addressing several health-related dimensions while taking into account the heterogeneity of older adults by providing a good characterization of the participants with respect to frailty.

## Appendix

Multimedia Appendix 1 PRISMA Checklist.

Multimedia Appendix 2 Search string.

Multimedia Appendix 3 Crossover vote.

Multimedia Appendix 4 Excluded articles based on full-text review.

Multimedia Appendix 5 Quantitative outcome data.

## Abbreviations

AT: assistive technology
CENTRAL: Cochrane Central Register of Controlled Trials
CINAHL: Cumulative Index to Nursing and Allied Health Literature
GRADE: Grading of Recommendations, Assessment, Development and Evaluations
PRISMA: Preferred Reporting Items for Systematic Reviews and Meta-Analyses
QoL: quality of life
RCT: randomized controlled trial
RoB: risk of bias
