# Supplementary material for: The Effectiveness of Assistive Technologies for Older Adults and the Influence of Frailty: Systematic Literature Review of Randomized Controlled Trials
Source: JMIR Aging. 2022 Apr 4;5(2):e31916. doi: 10.2196/31916 (PMC9016506; doi:10.2196/31916)
Supplement: Multimedia Appendix 2 [file aging_v5i2e31916_app2.pdf]

Multimedia Appendix 2: Search String (Ovid Syntax)

**Age**<br>Elder*.ti,ab,kf. OR (community adj1 dwelling).ti,ab,kf. OR geriatric.ti,ab,kf. OR mini-mental state.ti,ab,kf. OR alzheimer*.ti,ab,kf. OR mmse.ti,ab,kf. OR caregiver*.ti,ab,kf. OR falls.ti,ab,kf. OR adl.ti,ab,kf. OR frail*.ti,ab,kf. OR Gds.ti,ab,kf. OR Ag?ng.ti,ab,kf. OR Mci.ti,ab,kf. OR dement*.ti,ab,kf. OR (psycho-geriatric* OR psychogeriatric*).ti,ab,kf. OR cognitive impairment.ti,ab,kf. OR postmenopausal women.ti,ab,kf. OR comorbid*.ti,ab,kf. OR exp Nursing Homes/ OR Geriatric Assessment/ OR exp Aged/ OR Alzheimer disease/ep OR cognition disorders/di OR cognition disorders/ep OR Homes for the Aged/ OR disabilit*.ti,ab,kf. OR (functional adj2 (decline OR status)).ti,ab,kf. OR (gerontopsychiatry OR geronto-psychiatry).ti,ab,kf. OR activities of daily living.ti,ab,kf. OR exp Activities of daily living/ OR exp dementia/ OR old*.ti,ab,kf. OR disabled persons/ OR persons with hearing impairments/ OR visually impaired persons/ OR (mobil* OR immobil*).ti,ab,kf. OR Assisted Living Facilities/
**AND**
**RCT**<br>1 randomized controlled trial.pt.<br>2 controlled clinical trial.pt.<br>3 randomized.ab.<br>4 placebo.ab.<br>5 clinical trials as topic.sh.<br>6 randomly.ab.<br>7 trial.ti.<br>8 1 or 2 or 3 or 4 or 5 or 6 or 7<br>9 exp animals/ not humans.sh.<br>10 8 not 9
**AND**
**Technology**<br>((assist*.ti,ab. OR welfare.ti,ab. OR self-help.ti,ab. OR smart-home.ti,ab. OR home-based.ti,ab. OR safety.ti,ab. OR protective.ti,ab. OR intelligen*.ti,ab. OR health-care.ti,ab.) AND (technolog*.ti,ab. OR device*.ti,ab. OR platform*.ti,ab. OR solution*.ti,ab.)) OR assisted living.ti,ab. OR gerontechnolog*.ti,ab. OR device*.ti,ab. adj4 daily living.ti,ab. OR virtual reality.ti,ab. OR (mHealth.ti,ab. OR mobile health.ti,ab. OR pHealth.ti,ab. OR personal health.ti,ab.) OR exp self-help devices/ OR protective devices/
