# Supplementary material for: The Effectiveness of Assistive Technologies for Older Adults and the Influence of Frailty: Systematic Literature Review of Randomized Controlled Trials
Source: JMIR Aging. 2022 Apr 4;5(2):e31916. doi: 10.2196/31916 (PMC9016506; doi:10.2196/31916)
Supplement: Multimedia Appendix 3 [file aging_v5i2e31916_app3.pdf]

Multimedia Appendix 3: Crossover studies included in the Review: Vote regarding potential carry-over effects

| Author | Intervention | Trial period | Vote |
|---|---|---|---|
| Brath et al. | Mobile based electronic medication blisters vs regular blisters for patients with cardiovascular disease | 20 weeks, immediate crossover | **A 20-week long period of using electronic blisters, being reminded via SMS if data transmission was late and called by a study-coordinator if adherence sank to less than 70% can alter personal behavior. It is likely that this also influenced the control phase and caused better adherence with regular blisters.** |
| Bray et al. / Taylor et al. | Portable electronic vision enhancement system vs. regular vision aid for adults with moderate to severe vision impairment | 2 months, immediate crossover | **The device primarily helps while being used. Therefore, no serious carryover effect is expected.** |
| Davison et al. | Multimedia device with photographs, music, videos selected by relatives for people with dementia living in residential care | 4 weeks, immediate crossover | **Potential Positive effects of the device on depression or anxiety can carry over into the crossover phase and thus influence data collection at the end.** |
| Elston et al. | Metronome to improve mobility vs. regular medication in patients suffering from Parkinson's disease | 4 weeks, 6 week wash-out period | **The wash-out period probably helped to eliminate possible carryover effects. However, even the authors mention a potential carryover effect.** |
| Van der Ploeg et al. | Video chat (skype) vs. landline telephone to treat agitation in people with dementia | Four 20-min conversations within two weeks, immediate crossover | **Agitative behavior before and during the conversations was assessed. Only four conversations were analyzed in total. A serious carryover effect seems unlikely.** |
