# Supplementary material for: The Effectiveness of Assistive Technologies for Older Adults and the Influence of Frailty: Systematic Literature Review of Randomized Controlled Trials
Source: JMIR Aging. 2022 Apr 4;5(2):e31916. doi: 10.2196/31916 (PMC9016506; doi:10.2196/31916)
Supplement: Multimedia Appendix 5 [file aging_v5i2e31916_app5.pdf]

Multimedia Appendix 5: Quantitative data for primary outcome measures (Pre-test (baseline) and post-test for intervention and control groups, test statistic, effect size and significance level for between-group comparison)

Study (n[a])	Measure (Instrument) (range)			Mean (SD)				Results of statistical analysis		
				IG[b]		CG[c]		Test statistic[d]	Effect size	Significance level[e]
				Pre-test	Post-test	Pre-test	Post-test			
**Mobility**										
Elston et al [14] (n=42)	Parkinson's Disease Mobility (PDQ-39)			Crossover study with expected carryover (no data for first study phase)						
	QoL[f] (SF-36)									
Lauriks et al [6] (n=54)	QoL[f] as observed by caregivers (Qualidem)									
	Care relationship (0-21)			17.34 (3.73)	14.13 (4.50)	16.83 (3.59)	13.42 (4.93)	U = 336.5	0.05	*P*=.42
	Positive affect (0-18)			14.10 (3.30)	14.21 (2.54)	13.59 (3.63)	14.29 (3.2)	U = 337.5	0.2	*P*=.43
	Negative affect (0-9)			6.93 (2.12)	4.96 (2.26)	5.7 (2.64)	4.88 (2.85)	U = 309	0.52	*P*=.24
	Restless behavior (0-9)			5.28 (2.64)	5.21 (2.67)	5.31 (2.78)	4.38 (2.62)	F = 0.53	0.33	*P*=.24
	Positive self-image (0-9)			7.03 (2.24)	6.21 (2.41)	6.48 (2.13)	5.92 (2.75)	F = 0.02	0.13	*P*=.44
	Social relations (0-18)			12.59 (3.64)	12.42 (3.55)	12.41 (3.74)	11.75 (3.55)	U = 306	0.14	*P*=.22
	Social isolation (0-9)			7.07 (2.22)	6.21 (2.41)	7.28 (2.07)	5.29 (2.27)	F = 5.79	0.58	***P*=.01***
	Feeling at home (0-12)			9.66 (2.44)	8.42 (4.15)	9.03 (2.75)	7.58 (4.18)	U = 345.5	0.07	*P*=.48
	Having things to do (0-6)			2.62 (2.19)	2.96 (2.16)	3.14 (2.20)	2.58 (1.93	U = 230.5	0.62	***P*=.02***
	self-rated dementia QoL[f] (DQoL) (30-150)			102.69 (10.63)	109.67 (7.98)	100.57 (7.7)	100 (13.89)	U = 25	0.64 (Cohen's d)	*P*=.15
	Need for support and care of people with dementia (CANE)									
	Housing	No need		n = 0	n = 0	n = 0	n = 0	could not be calculated		
		Met need		n = 23	n = 23	n = 14	n = 14			*P*=1
		Unmet need		n = 0	n = 0	n = 0	n = 0			
		Satisfaction with support	Yes	n = 20	n = 22	n = 12	n = 12	χ^2 = 1.29		*P*=.13
			No	n = 3	n = 2	n = 1	n = 2			
	Memory	No need		n = 0	n = 1	n = 2	n = 2	χ^2 = 1.86		
		Met need		n = 23	n = 22	n = 12	n = 12			*P*=.2
		Unmet need		n = 0	n = 0	n = 0	n = 0			
		Satisfaction with support	Yes	n = 20	n = 13	n = 22	n = 14	χ^2 = 2.26		*P*=.16
			No	n = 0	n = 1	n = 1	n = 0			
	Mobility	No need		n = 3	n = 2	n = 3	n = 2	χ^2 = 0.67		
		Met need		n = 20	n = 21	n = 11	n = 12			*P*=.36
		Unmet need		n = 0	n = 0	n = 0	n = 0			

	Satisfaction with support – Yes	n = 23	n = 22	n =13	n = 14	χ^2 = 2.26		*P*=.16
	Satisfaction with support – No	n = 0	n = 1	n = 1	n = 0			
	Psych. distress – No need	n = 13	n = 12	n = 6	n = 4	χ^2 = 2.9		
	Psych. distress – Met need	n = 7	n = 9	n = 6	n = 8			*P*=.2
	Psych. distress – Unmet need	n = 3	n = 2	n = 2	n = 2			
	Psych. distress – Satisfaction with support – Yes	n = 20	n = 21	n = 12	n = 12	χ^2 = 1.99		*P*=.19
	Psych. distress – Satisfaction with support – No	n = 3	n = 2	n = 2	n = 2			
	Total fall incidents		54 (13)[i]		83 (16)[i]	U = 275.5	0.35 (Cohen's d)	*P*=.06
	Total number of use of restraints		4 (3)[i]		7 (4)[i]	U = 350.5	0.11 (Cohen's d)	*P*=.48
	Caregiver job satisfaction (MJS-HC) (1-5)	3.79 (0.43)	3.8 (0.42)	3.7 (0.42)	3.64 (0.23)	F = 0.46	0.17 (Cohen's d)	*P*=.25
	Caregiver workload (MBI, Dutch version)							
	Workload experience (1-5)	2.71 (0.47)	2.57 (0.51)	2.64 (0.51)	2.36 (0.67)	U = 66.5	0.19 (Cohen's d)	*P*=.26
	Skip breaks due to workload (1-4)	2.07 (0.62)	1.86 (0.77)	1.64 (0.67)	1.91 (0.94)	F = 0.01	-0.4 (Cohen's d)	*P*=.27
	Increase due to pers. shortage (1-5)	1.5 (0.86)	1.45 (0.93)	1.45 (0.93)	1.82 (1.17)	F = 0.15	-0.22 (Cohen's d)	*P*=.28
	work circumstance appreciation compared to three months earlier (pre-test) (1-7)	5.5 (1.23)	4.64 (1.15)	5.55 (1.29)	5.45 (1.13)	F = 5.02	-0.51 (Cohen's d)	***P*=.02***
	Caregiver general health (GHQ-28) (0-84)	9.93 (3.85)	11.08 (4.43)	9.27 (2.24)	11.91 (5.99)	F = 0.58	0.23 (Cohen's d)	*P*=.29
Scheffer et al [52] (n=203)	Frequency of going outside (baseline in summer, post-test in winter)							
	daily/weekly (% of participants)	93.8%	77.9%	93.2%	82.6%			
	monthly/never (% of participants)	6.1%	22.1%	6.8%	17.4%			
Schoon et al [53] (n=86)	Subjective general health and mental wellbeing (MOS-20) (0-100)							
	Physical functioning	39.9 (31.9)	Δ = -2.8 (25)	44.6 (32.9)	Δ = -1.2 (26.4)			*P*=.411
	Role functioning	27.9 (41.3)	Δ = 11.1 (38)	52.3 (47.5)	Δ = -4.8 (39.5)			*P*=.776
	Social functioning	60 (33.5)	Δ = -3.9 (22.3)	72.1 (28.3)	Δ = -1.4 (25.9)			*P*=.64
	Mental health	70.2 (21)	Δ = -2 (11.3)	72.7 (20.8)	Δ = -0.8 (11.4)			*P*=.496
	Current health perception	58.8 (25.0)	Δ = -1.3 (17.1)	59.1 (23)	Δ = -3.9 (16.4)			***P*=.024***
	Pain	41.7 (34.8)	Δ = 7.1 (26.8)	47.7 (33.1)	Δ = -2.4 (40.5)			*P*=.587
	Number of weekly measurements of gait speed (compliance)		82.1% (57.6, 100)[j]					
	Fall incidence		0.9 (1.8)		1.9 (4.1)	*t*(57.7) = 1.4		*P*=.155
	Incidence of injurious falls		1.4 (1.5)		1.7 (2.4)	*t*(34) = 0.5		*P*=.637
	Fear of Falling (FES-1) (16-64)	32.8 (11.4)	Δ = 2.1 (6.9)	31 (9.7)	Δ = -1.6 (7.0)	F(1,69) = 2.6		*P*=.110

Tchalla et al [7] (n=96)	Fall incidence		32.7%		63.8%		OR = 0.37	**95%CI = 0.15 to 0.88***
**Personal disease management**								
Hägglund et al [54] (n=82)	Heart failure self-care behavior (EHFScB-9) (9-45)	26 (17,29)[j]	17 (13, 22)[j]	23 (15,31)[j]	21 (17, 25)[j]			***P*<.05***
	Health-related QoL[f] (KCCQ) (0-100)	50 (22.9, 62)[j]	65.1 (38.5, 83.3)[j]	42.7 (21.9, 51.6)[j]	52.1 (41.1, 64.1)[j]			***P*<.05***
Levine et al [48] (n=54)	Glycated hemoglobin level (HbA1c (%))	8.13%	7.53%	7.57%	7.76%	No numbers reported for between group comparison. Within group comparison showed a significant decrease only in the IG[b]: F(1,20) = 4.63, η^2_p = 0.188, p = .044.		
	Frequency of self-monitoring of blood glucose		6% missed measures		>22% missed measures	$\chi^2(1)$ = 977.53		***P*<.001***
Ong et al [51] (n=197)	Emergency department visits	1 (1, 2)[j]	0 (0, 1)[j]	1 (1, 2)[j]	0 (0, 1)[j]			*P*=.881
	Number of hospitalizations	1 (0, 1)[j]	0 (0, 1)[j]	1 (0, 2)[j]	0 (0, 1)[j]			*P*=.545
	Total length of stay for admitted patients (in days)		8 (4, 14)[j]		15 (3, 25)[j]			***P*=.045***
Or and Tao [13] (n=63)	Glycated hemoglobin level (HbA1c (%))	7.4 (1.2)	7.2 (6.7 to 7.8)[k]	7.3 (0.76)	7 (6.4 to 7.7)[k]			
	Fasting blood glucose level (mmol(dL))	8 (3.1)	7.1 (5.3 to 8.8)[k]	7.7. (3.4)	8.1 (6.1 to 10)[k]			*P*=.407
	Systolic blood pressure (mm Hg)	134.9 (130 to 139.8)[k]	121.9 (116.9 to 126.9)[k]	130.1 (124.8 to 135.4)[k]	124.6 (119.3 to 130)[k]			***P*=.043***
	Diastolic blood pressure (mm Hg)	79.5 (76 to 82.9)[k]	73.8 (70.2 to 77.3)[k]	76.1 (72.4 to 79.9)[k]	74.2 (70.4 to 78)[k]		Significant within-group change in the IG[b]: Δ = -5.7 (95% CI = -9.3 to -2.20)	
	Diabetes knowledge (% of correct responses)	37.3 (27.9 to 46.7)[k]	44.1 (34.5t o 53.7)[k]	43.4 (32.8 to 54)[k]	51.5 (40.9 to 62.1)[k]			
	Hypertension knowledge (% of correct responses)	49.3 (44.4 to 54.1)[k]	51.8 (46.8 to 56.7)[k]	50.2 (44.9 to 55.4)[k]	51.8 (46.5 to 57.1)[k]			
	Self-monitoring frequency (number of times per week)							
	Patients with hypertension (blood pressure)		8 (4.2)		8.6 (3.4)			*P*>.05
	Patients with diabetes mellitus (blood glucose)				4.3 (1.4)			*P*>.05
	Patients with hypertension and diabetes mellitus (blood pressure, blood glucose)		6.1 (3.5), 3.5 (2.7)		7.1 (2.4), 5.5 (1.8)			*P*>.05

Rantz et al [22] (n=171)	Walking speed (in seconds/10ft)	7.6	7.64	7.6	8.4	F(113) = 2.23		*P*=.1384
	GaitRite[g] Functional Ambulation Profile (30-100)	66.8	64.84	67.3	61.61	F(112) = 3.14		*P*=.0792
	QoL[f] (SF-36)	No numbers reported. Values were not statistically significant.						
	Depression (GDS)							
	Mental State (MMSE)							
	ADL[h]							
	IADL[h]							
	Hand grip							
**Medication**								
Brath et al [27] (n=77)	Medication adherence	Crossover study with expected carryover (no data for first study phase)						
Goldstein et al [17] (n=60)	Medication adherence (devices with reminders (IG[b]) vs. devices without reminders (CG[c]))		79% (36)		78% (33)			*P*=.87
Lam et al [12] (n=134)	Self-efficacy for appropriate medication use (SEAMS) (13-39)	31.4 (7.19)	32.76 (6.71)	32.24 (6.21)	33 (5.38)	*t*(109) = 0.21		*P*=.838
	Medication adherence (MMAS-8) (0-8)	6.5 (1.67)	7.06 (1.27)	6.63 (1.52)	7.07 (1.25)	*t*(108) = 0.46		*P*=.496
	Refill adherence (CMG)		6.57 (7.93)		8.85 (11.71)	*t*(127) = 1.30		*P*=.197
	Medication knowledge (percentage of correctly answered questions)	0.82 (0.13)	0.93 (0.09)	0.85 (0.12)	0.93 (0.11)	*t*(159) = -0.07		*P*=.948
	Systolic blood pressure	142.98 (21.05)	138.89 (19.37)	142.16 (17.23)	139.63 (15.29)	*t*(127) = 0.24		*P*=.810 Significant within-group change in IG[b]: t(64 = -2.14; *P*=.036
	Diastolic blood pressure	77.13 (13.16)	74.71 (13)	75.84 (11.89)	75.23 (10.52)	*t*(127) = 0.25		*P*=.801 Significant within-group change in IG[b]: t(64) = -2.27; *P*=.027
Mira et al [50] (n=102)	Self-perceived health status	71.27 (17)[l]	74.6 (17)[l]	68.3 (21)[l]	69.1 (20)[l]		Δ = 1.2	*P*=.54
	Medication adherence (MMAS-4)	6.6 (1.2)	7.4 (0.9)	7.2 (0.9)	7.3 (0.7)		Δ = 0.7	***P*<.001***
	Medication errors							
	0 errors in the three months before	38 (74)[l]	43 (84)[l]	42 (87)[l]	43 (90)[l]			*P*=.95
	1 error in the three months before	9 (18)[l]	6 (12)[l]	6 (12)[l]	3 (6)[l]			
	2 or more errors in the three months before	4 (8)[l]	2 (4)[l]	0 (0)[l]	2 (4)[l]		Δ = 0.2	***P*<.001***

	Number of missed doses					27.3% reduction in missed doses in IG[b], no significance levels reported.		
**Mental support**								
Davison et al [11] (n=16)	Agitation (Cohen Mansfield Agitation Inventory)	Crossover study with expected carryover (no data for first study phase)						
	Depression in Dementia (CSDD)							
	Anxiety in Dementia (RAID)							
Van der Ploeg et al [8] (n=17)	Agitation (Cohen-Mansfield Agitation Inventory)		Δ = -24.1		Δ = -12.9	*t*(8) = 1.208		*P*=.262
	Call duration (in minutes)		12		10.3	*t*(8) = -1.992		*P*=.082
**Hearing**								
Adrait et al [23], Nguyen et al [55] (n=51)	Neuropsychiatric symptoms (NPI)	17.5 (12.3)	23.6 (22.6)	25.8 (15.1)	26.1 (14.7)			*P*=.3
	IADL[h]	4.7 (2.1)	3.2 (2)	4 (2.5)	3 (2.3)			*P*=.6
	Alzheimer's Disease related QoL[f]	457.4 (112.4)	452 (88.4)	450.4 (81.2)	446.4 (45.8)			*P*=.2
	Caregiver QoL[f] (Zarit)	19.6 (10.6)	22.4 (14.7)	26 (14.4)	26.3 (15.2)			*P*=.5
	Duke health profile patient	4.5 (2)	3.9 (2.3)	3.7 (1.4)	4.8 (1.9)			*P*=.3
	Duke health profile caregiver	4.6 (2.3)	4.6 (1.8)	3.9 (2)	4.3 (1.8)			*P*=.6
	Alzheimer's Disease Cognition (ADAS-Cog) (0-70)	18.1 (7.4)	20.4 (8.1)	19 (9.5)	19.3 (10.1)			*P*=.8
Humes et al [49] (n=163)	Global hearing aid performance and benefit (PHAPglob)<br>AB: audiology-based (best-practice)<br>CB: consumer-decides	AB: 0.36 (0.12)<br>CB: 0.38 (0.13)	AB: 0.17 (0.12)<br>CB: 0.12 (0.12)	0.4 (0.13)	0.04 (0.1)	F(2,160) = 20.31		***P*<.001***
**Vision**								
Bray et al [24], Taylor et al [56] (n=100)	Near vision visual function (NV-VFQ-15)					*t*(67.3) = 4.69	Δ = 0.57	**95% CI = 0.33 to 0.81***
	Vision-related QoL[f] (VisQoL) (0-1)						Δ = 0.01	**95% CI = -0.02 to 0.05***
	Cost-effectiveness (NV-VFQ vs. carer & intervention costs)							
	Cost utility (cost per quality adjusted life year based on VisQoL)							
	Maximum reading speed for high contrast sentences (MRS)					*t*(70.71) = 1.18	4.04	95% CI = -2.81 to 10.9
	Frequency of use (MLVQ) (0-4)					*t*(75.69) = -5.19	-0.93	**95% CI = -1.29 to -0.57***

[a]Number of participants randomized
[b]IG: Intervention group
[c]CG: Control group

[d]Test statistic from t-Test (t), ANOVA/ANCOVA (F), Mann-Whitney U-Test (U), regression analysis (OR: Odds Ratio) or Chi-Square test (χ^2); degrees of freedom in brackets.
[e]Significance levels reported as 95% Confidence Intervals (CI) or P-values
[f]QoL: Quality of Life
[g]Automatic measurement of certain variables (e.g., velocity, step length) while participants walk across the GAITRite Mat
[h](I)ADL: (Instrumental) Activities of daily living
[i]Total incidents (involved n)
[j]Median (Inter quartile range)
[k]Mean (95% CI)
[l]n (%)
*Statistical significance ($p < .05$)
